# A liposomal Gd contrast agent does not cross the mouse placental barrier

**DOI:** 10.1038/srep27863

**Published:** 2016-06-14

**Authors:** Anil N. Shetty, Robia Pautler, Ketan Ghagahda, David Rendon, Haijun Gao, Zbigniew Starosolski, Rohan Bhavane, Chandreshkumar Patel, Ananth Annapragada, Chandrasekhar Yallampalli, Wesley Lee

**Affiliations:** 1 Department of Obstetrics and Gynecology, Texas Childrens Hospital, 6621 Fannin Street, Houston, TX 77030, USA; 2 Department of Molecular Physiology and Biophysics, Baylor College of Medicine, 1 Baylor Plaza, Houston, TX 77030, USA; 3 Department of Pediatric Radiology, Texas Children’s Hospital, Houston, Texas

## Abstract

The trans-placental permeability of liposomal Gadolinium (Gd) nanoparticle contrast agents was evaluated in a pregnant mouse model. Pregnant Balb/c mice at 16.5 (±1) days of gestation were imaged using a 3D Spoiled Gradient Echo method at 9.4 T using two contrast agents: a clinically approved Gd chelate, Multihance^®^ (gadobenate dimeglumine), and a novel experimental liposomal Gd agent. A Dynamic Contrast Enhancement (DCE) protocol was used to capture the dynamics of contrast entry and distribution in the placenta, and clearance from circulation. A blinded clinical radiologist evaluated both sets of images. A reference region model was used to measure the placental flow and physiological parameters; volume transfer constant (K^trans^), efflux rate constant (K^ep^). The Gd content of excised placentae and fetuses was measured, using inductively coupled plasma mass spectrometry (ICP-MS). MRI images of pregnant mice and ICP-MS analyses of placental and fetal tissue demonstrated undetectably low transplacental permeation of the liposomal Gd agent, while the clinical agent (Multihance) avidly permeated the placental barrier. Image interpretation and diagnostic quality was equivalent between the two contrast agents. Additional testing to determine both maternal and fetal safety of liposomal Gd is suggested.

Efficient exchange of nutrients between the mother and the fetus critically depends upon the uteroplacental and fetoplacental circulations^1^,^2^,^3^,^4^. Placental under-perfusion could contribute to fetal growth restriction, and its measurement could serve as a potential predictor of adverse pregnancy outcomes^2^,^4^. Undetected structural anomalies of the placenta such as placenta accreta, invasion of the placenta into the uterine wall, result in massive obstetric hemorrhage at childbirth. However, our current ability to assess placental structure and function is limited by a lack of techniques that are safe in pregnancy. Ultrasound followed by MRI has a ~85% sensitivity for placenta accreta. Ultrasonography and Doppler methods for functional evaluation have limited success, and have poor predictive value for adverse pregnancy outcomes^5^,^6^. Contrast-enhanced ultrasound using micro-bubbles as tracers has been applied but is dependent on the region of interest and remains highly operator dependent^1^,^3^,^7^. Radiolabeled microspheres and angiography have been used for measuring placental perfusion in animals but safety concerns due to radiation exposure preclude their use in human pregnancy^2^,^4^,^8^,^9^.

Contrast enhanced MRI is well suited to the assessment of both structure and perfusion dynamics, thus permitting detailed characterization of tissues and vascular structures. However, the use of contrast agents in human gravidae is discouraged because the placental-fetal membrane is permeable to most contrast agents, engendering potential exposure of the fetus. The technique has been demonstrated in animal models^5^,^6^,^10^,^11^ and can be used to quantitatively estimate permeability characteristics^12^,^13^.

We tested two agents. (1) Gadobenate dimeglumine (Multihance^®^), a clinically used Gadolinium chelate, with a relatively short circulation half life and practically complete renal excretion. Like many Gd chelates, this agent does not significantly bind serum proteins, has a finite permeability through vascular endothelium, and its distribution volume is roughly equal to total extracellular water^14^,^15^,^16^. Gadolinium induced toxicity primarily results from the dissociation of free Gd from the chelate, and to a relatively small extent from renal toxicity of intact chelate. Gadobenate is among the most stable chelates, and has been shown to release no more free Gd in native human serum^17^,^18^ than the two other well known low risk agents gadofosveset trisodium (Ablavar^®^) and gadopentetate dimeglumine (Magnevist^®^). However, of these three agents, the two linear chelates gadobenate and gadopentetate have relatively low albumin binding. Human serum albumin has significant permeability through the placental membrane^19^ and could therefore transport Gd across the barrier if it were to bind the Gd chelate. We therefore chose gadobenate dimeglumine as the conventional agent to test.

(2) A novel liposomal contrast agent, encapsulating gadobenate dimeglumine in the interior, and incorporating a lipid-Gd-Chelate conjugate in the bilayer. Due to their large size (100–150 nm diameter, molecular weight ~ 2 × 10^5 ^kD), lipsomes exhibit very limited diffusion through intact vascular endothelium^20^. The liposome based Gd agents used in this study remain in the blood pool with a half-life of 18 to 24 hours. They are eliminated from blood by the reticulo-endothelial system and cleared by the liver and spleen^21^,^22^.

We hypothesized that nanoparticle based contrast agents would (1) exhibit reduced permeation of the placental barrier compared to conventional agents, (2) demonstrate placental anatomy at least as well as conventional agents without toxicity to fetus.

## Results

### Mouse DCE-MRI studies

The measured relaxivity (r1) of Multihance^®^ was 3.99 ± 0.52 and liposome Gd was 2.50 ± 0.29 (sec·mM)^−1^ at 9.4 T. (Supplemental Fig. S1). *In vivo*, the pre-contrast relaxation time (T_1_(0)) ranged from 800–1100 ms. The number of placentae visible varied from 2 to 5 per animal. The fetal-placental sac, placenta and fetus were distinguishable on T_1_-weighted post-contrast images. The maternal artery and fetal left ventricle were not visible either due to restricted field-of-view (FOV) or from motion related blurring. Of the 25 mice tested, 14 mice received Multihance^®^ and the images yielded 52 analyzable placentae. 9 mice received liposomal Gd and the images yielded 36 analyzable placentae. 2 mice received saline placebo and the images yielded 4 analyzable placentae.

**Figure 1:**
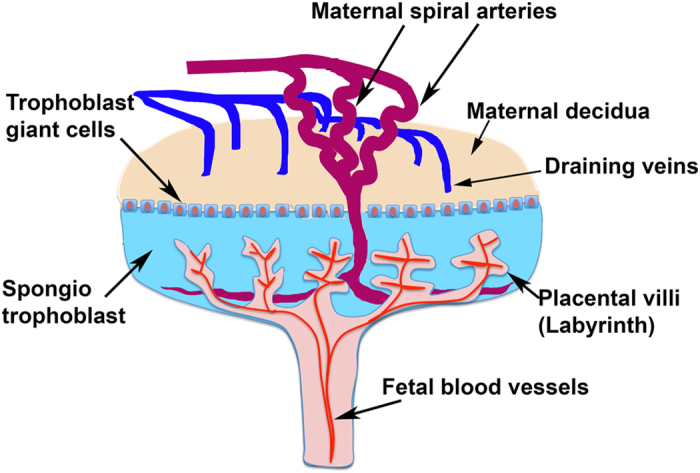
Structure of the mouse placenta. The mature placenta (E14.5) consists of three layers: the labyrinth, the spongiotrophoblast, and the maternal decidua. The inner compartment of the hemochorionic placenta - the labyrinth - contains the villi where nutrients pass from the maternal blood into the foetal blood.

Figure 1 shows a diagram of the placental circulation, and the locations of the labyrinth and decidual zones, as used in this analysis. The union of the labyrinth zone and the decidual zone was considered to be the entire placenta. Supplemental movie Movie 1 shows the results of a typical DCE-MRI using gadobenate dimeglumine contrast. 4 placentae are seen enhanced by contrast, as the bolus moves into them. The labyrinth zone receives the contrast agent first, followed by a gradual filling of the rest of the placenta, further followed by a gradual decay in intensity as the agent is cleared from the maternal circulation. At the same time, a slight increase in intensity in the fetal sac is seen, as the agent filters into the fetal compartment.

**Figure 2:**
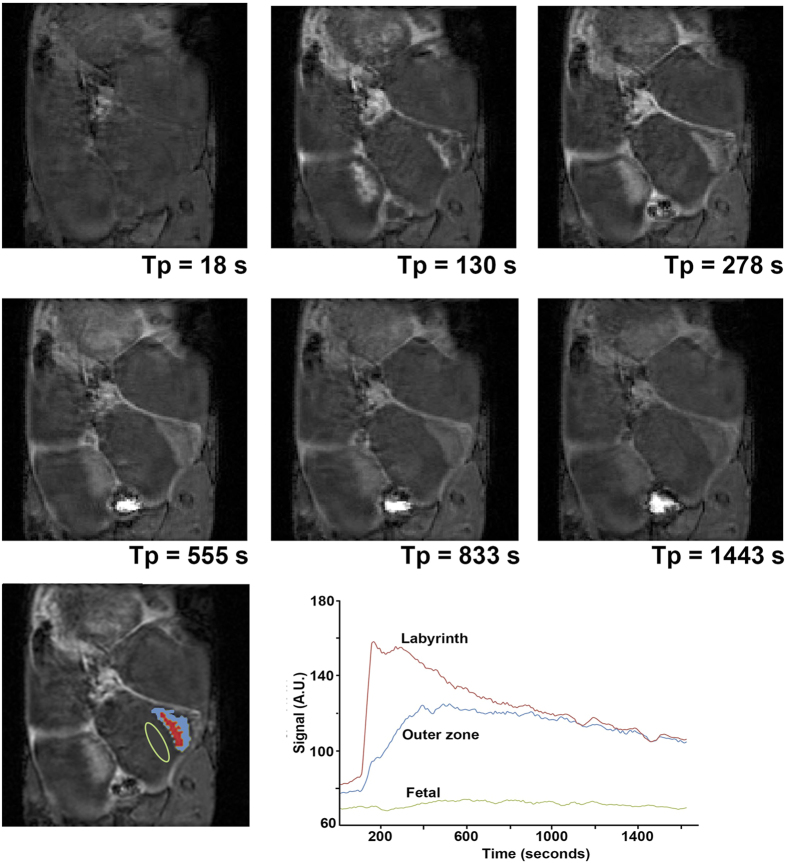
Dynamic signal enhancement during the entry of conventional contrast agent (Multihance^®^:gadobenate dimeglumine) into the placenta. At 130 seconds, an artery carrying maternal blood to the placenta, the central arterial canal, and placental labyrinth are all enhanced. At subsequent points, the details of placental vasculature are obscured by enhancement of the peripheral placenta. The placental labyrinth shows early enhancement (red) followed by the periphery (blue). Both zones then fade as the contrast is cleared from circulation. The transient signal in the two enhancing zones is shown in the graph.

**Figure 3:**
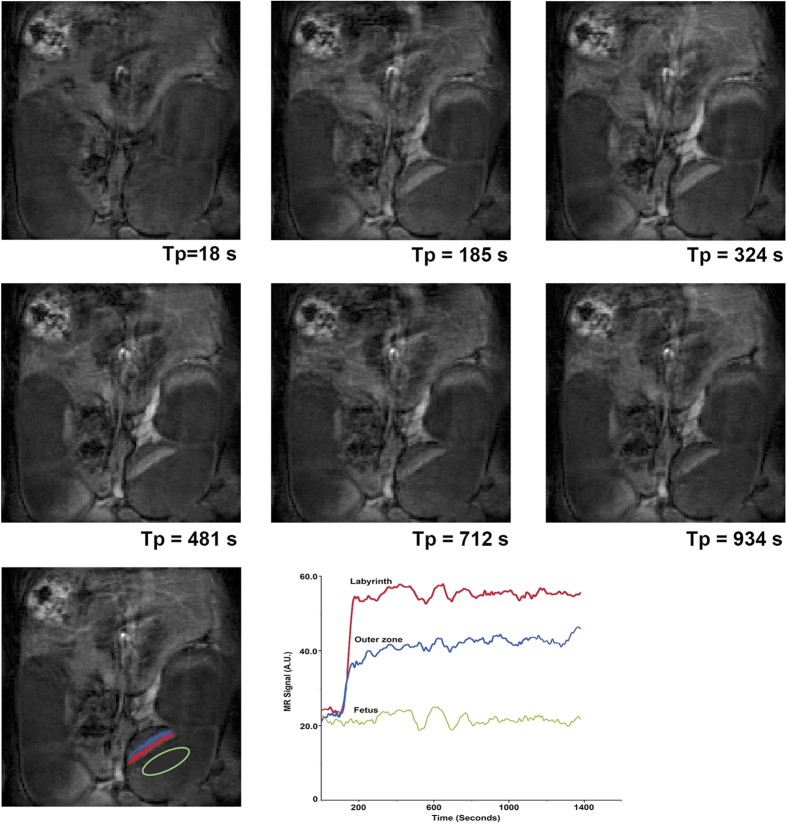
Dynamic signal enhancement during the entry of liposomal contrast into the placenta. At 18 seconds, pre contrast entry, there is no enhancement visible. At 185 seconds, vessels entering the placenta, the labyrinth and periphery, are all enhanced. As the enhancement slowly fades, the labyrinth appears to retain contrast even at the last time point collected (1221 seconds, ~20 minutes post injection).

The signal enhancement at progressive time points, as a result of contrast-agent circulation is shown in Fig. 2. The central arterial canal feeding in to the placental labyrinth is visible at the 130 s time point. Within each placenta, two zones of flow are seen (the placental labyrinth, and the maternal decidua or peripheral region) as shown with color highlighting in the bottom left. The placental labyrinth fills first and reaches peak enhancement before the proximal decidua peaks in intensity. Figure 3 shows the same transient filling sequence when using the liposomal Gd contrast agent. While gadobenate dimeglumine shows a distinct delay between the filling of the placental labyrinth and the maternal decidua, no such delay is noted with the liposomal contrast agent.

Table 1 shows the objective impressions of a blinded radiologist who read the images from each contrast agent, at peak intensity, and assessed each placenta for clarity of visualization. The blinded reviewer reviewed all 52 placentae visible in the 14 mice treated with conventional Gd (Multihance) and all 36 placentae visible in the 9 mice treated with liposomal Gd. The χ^2^ test yielded a probability of 2 × 10^−8^ that the two score distributions were different, suggesting that the two contrast agents enabled images of the same diagnostic quality.

**Figure 4:**
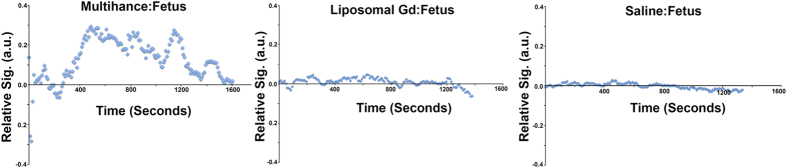
Typical signal intensity in the fetus, following conventional, liposomal and blank (saline) contrast injections. (**A**) Multihance^®^ injection shows uptake in the fetal compartment; (**B**) liposome Gd shows no visible uptake, indistinguishable from the saline blank injecton shown in (**C**).

In every mouse studied, uptake of contrast agent in the fetal compartment was observed with gadobenate dimeglumine whereas with liposomal Gd, no uptake was seen in the fetal compartment even after 72 hr exposure. Figure 4 shows representative fetal signal following injection of each of the two contrast agents and a control saline injection. The image data was used to calculate absolute Gd concentrations using the methods in Appendix A. Contrast agent concentrations estimated from the MRI data are shown in Table 2 using Eqs. A4 and A5.

**Figure 5:**
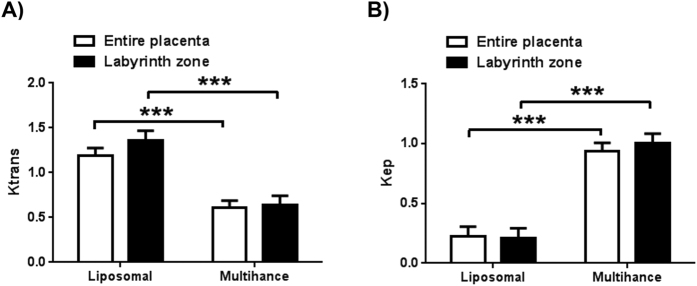
Pharmacokinetic (PK) parameters obtained using the RR model. Transfer rates are based on assuming ktrans = 0.2 and Kep = 0.1 for para-spinal reference tissue. The RR model analysis was performed in two ways: (1) by using signal from the entire placenta as a single compartment (shown in graphs as a white bar) and (2) using only the central labyrinth as an early enhancement signal compartment (black bar). The transfer rates from maternal arteries to placental vascular compartment is Ktrans (shown in (**A**)), the efflux rate for placental vascular compartment output is Kep (shown in (**B**)). The statistical significance of difference between the measurements two agents is marked using the probability p testing the null hypothesis H0: Compared values are equal. The symbol ***represents p < 0.001. For each agent there was no statistically significant difference in PK parameters computed over the entire placenta vs the labyrinth alone. The error bars represent standard error of the mean.

K^trans^ and K^ep^ values for each contrast agent, and considering either the labyrinth zone or the entire placenta as the target tissue, are shown in Fig. 5. The values do not vary significantly with the location of the target ROI within the placenta, but reflect very different values for the two contrast agents.

**Figure 6:**
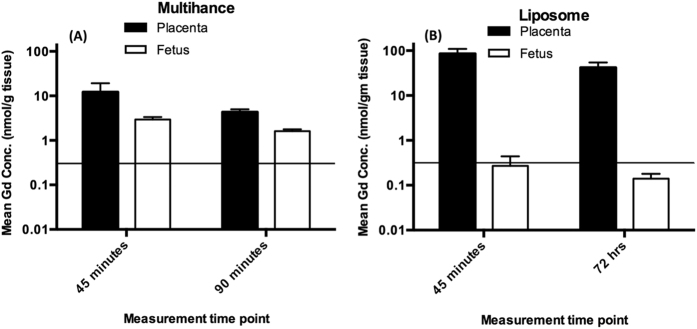
Gadolinium assay using ICP-MS, in placentae and fetuses from animals injected with Multihance^®^ (A; n = 8) and liposome Gd (B; n = 6). Gd concentration is presented at two time-points (45 minutes and 90 minutes post injection for Multihance^®^, 45 minutes and 72 hours for liposomal Gd). The detection limit is 0.318 nmol/g tissue and is shown by horizontal line. The error bars represent standard error of the mean. Note that the ordinate is plotted on a logarithmic scale, in order to insure visibility of the otherwise very low fetal concentrations following injection of liposomal Gd. Two fetal samples at the 45 minute time point following liposomal Gd injection yielded a Gd measurement above the detection limit, however, it is not possible to rule out the possibility of a small amount of placental tissue contaminating the fetal tissue during excision. All other liposomal Gd samples were below the detection limit. All samples following Multihance^®^ injection were above the detection limit.

Gadolinium assay by ICP-MS was performed on tissue samples collected at 45 min, 90 min and 72 hrs post-injection. The results of this analysis are shown in Fig. 6. Placental and fetal concentrations were within an order of magnitude of each other at 45 minutes, and within a factor of 2 of each other by 90 minutes when gadobenate dimeglumine was used. When the liposomal agent was used, the fetal concentrations are below the detection limit of the instrument (horizontal line on graph), and at least 2–3 orders of magnitude lower than the placental concentration. The Gd concentration in the fetus resulting from liposomal Gd injection was actually indistinguishable from the negative control (saline treated) control mice at both time points (data not shown).

## Discussion

The short gestational term of the mouse makes the mouse an attractive model for studying placental pathologies throughout the gestational period. The gestational age of mice in this study, at E16.5 ± 1 days, corresponds to the early part of the third trimester in humans. Both the human placenta and the mouse placenta are hemochorionic. There are structural differences, for example the maternal-fetal exchange zone is labyrinthine in mice, while it is villous in humans. However, these structural differences are considered to not affect the overall mechanism of exchange between utero-placental and feto-placental blood^23^,^24^.

The placenta is an organ of exchange, and therefore one expects selective, facile transport of species across the placental barrier. Our observation of fetal Gd levels being about 3 orders of magnitude lower than placental levels when liposomal Gd was used, compared to practically equal levels (within an order of magnitude) when conventional Gd chelate was used, (Fig. 6) is consistent with the liposomal particles having a far lower diffusion rate through the maternal-fetal barrier in the placenta. Active transport of liposomes across the placental barrier can be reasonably ruled out, because the PEG coating on the liposomes effectively prevents any specific binding to the surface. In the absence of an active transport mechanism, diffusive or convective processes are the only remaining mechanisms by which molecules or particles can be transported across the placental barrier. There is no convective flow across the barrier of an intact placenta, leaving diffusion as the sole mechanism. The liposomes being 3 orders of magnitude larger than free molecules, it stands to reason they must diffuse far slower, even in bulk medium. In the restricted geometry of the placental barrier, this difference in diffusion rates is likely to be even greater.

Our observation of two zones within each placenta with distinct temporal enhancement patterns is consistent with the early enhancing central labyrinth zone and late enhancing junctional and peripheral zones as previously described by Remus *et al*.^25^. The maternal blood enters the placenta via the penetrating arteries that focus into the central arterial canal (Fig. 1) and flows into the peripheral zone (the labyrinthine sinusoids, which are near the chorionic plate on the fetal side of the placenta). Then, the direction of flow reverses so that it travels away from the chorionic surface and towards the junctional zone^26^. While the two zones do not represent anatomically distinct regions, they can be segmented based on the dynamic signal enhancement pattern. The delivery of blood in this fashion, to the placental labyrinth via the central arterial canal, and its return via the extensive venous circulation, is demonstrated in the DCE-MRI images (Figs 2 and 3).

Clear delineation of the placental margin from the uterine wall is a crucial component of the diagnosis of placenta accreta and its related conditions (increta, percreta). Such delineation is a significant challenge today because it is not easy to visualize the thin tissue border that separates the placenta from the maternal uterine wall. Current practice utilizes ultrasound as the first method of visualization, with unclear cases being referred to MRI. No contrast agent is used for MRI in order to eliminate potential exposure of the fetus to Gadolinium. Sensitivity for detection of placental abnormalities using this algorithm is modest, between 80 and 85%^27^. In the absence of contrast, T1 weighted imaging results in poor conspicuity of the placental margin. The benefit of conventional T2-weighted imaging in the absence of exogenous contrast agents is unclear^28^,^29^. Tanaka *et al*.^30^ noted these difficulties with using MRI for the diagnosis of placenta accreta observing that it was not possible to differentiate chorionic villi from decidua basalis. With exogenous contrast however, clear differentiation was observed. Advanced MR techniques such as arterial spin labeling (ASL) and Diffusion Weighted Imaging (DWI) may overcome these limitations using endogenous contrast between tissue and blood^31^. ASL methods provide information on regional flow, without differentiating between the maternal and fetal side of circulation, while DWI provides information on the local diffusion coefficient of protons by measuring intravoxel incoherent motion. However, the Signal to Noise Ratio (SNR) and temporal resolution of both these techniques are significantly worse than contrast enhanced MR. Our observation that liposome Gd agents do not appear to cross in to fetal compartment, suggests a reduced risk to the fetus Their use could enable the visualization of placental boundaries with increased contrast, and the degree of placental invasion could potentially be determined with increased confidence^27^.

SNR in contrast enhanced MRI is a function of both the relaxivity of the contrast agent and the field strength, and increases with both these parameters^32^. The liposomal contrast agent used in this work, however, exhibits dramatically higher relaxivity at low field strength, consistent with the slower rotational correlation time of the liposomal agent compared to the Gd chelate in solution^32^,^33^. Thus, while the present work was conducted using a small animal high field 9.4 T magnet, equivalent or superior image quality is anticipated when using clinically relevant 1.5 T field strength.

Beyond simple margin delineation, Dynamic Contrast Enhancement can provide direct information about the net transport of contrast agent in the placenta. Chalouhi *et al*.^34^ and Taillieu *et al*.^35^, measured trans-placental permeability and placental perfusion using low-molecular conventional contrast agent and a three-compartment model (SAAM II). However, nearby arteries were difficult to visualize in the observed field-of-view and could not provide an accurate vascular input function. We therefore chose the Reference Region (RR) model^36^,^37^ approach that is not dependent upon a knowledge of arterial or vascular input function.

K^trans^ values in the RR model reflect the filling (perfusion) of the target tissue by the vascular contrast, while the K^ep^ values represent the elimination of contrast. We observe K^trans^ values for the liposomal contrast moderately higher than those for gadobenate dimeglumine, while K^ep^ values for the liposomal contrast are substantially lower than those for gadobenate dimeglumine. The low molecular weight gadobenate dimeglumine diffuses readily past the placental barrier, and therefore reduces the effective amount of contrast in the placenta itself, resulting in the lower K^trans^ value. Similarly, the faster clearance of the low molecular weight chelate by renal filtration results in a higher K^ep^ value compared to that for the liposomal Gd that clears rather slowly due to reticulo-endothelial system uptake and biliary elimination.

### Potential sources of error and study limitations

The potential uptake of liposomes by trophoblast cells has not been considered in this work, and is technically possible. If this were to occur, the effect of such internalization on trophoblast function will have to be considered, and the kinetics of perfusion as modeled by the two compartment approximation will have to be reconsidered. However, there is evidence for the lack of uptake of PEGylated liposomes by trophoblasts, and for that matter by all placental cell types, in the context of liposomes as a way of delivering drug to maternal disease sites while sparing the fetus^38^,^39^.

The ICP-MS measurement yielded the net concentration detected in each compartment. The mean concentration of Gd in the fetal compartment when the liposomal contrast agent was used, was below the detection limit of the ICP assay. Two individual measurements however, yielded detectable amounts of Gd in fetus but it is not possible to rule out contamination while extracting the fetus and the inadvertent inclusion of a small amount of placental tissue in the fetal sample. The data is consistent with predominant non-transport of the liposomal Gd across the placental barrier, as suggested by the perfused organ study.

The use of a 3D pulse sequence technique to acquire multiple slices covering a larger anatomy compromises temporal resolution, although this is mitigated by the ability to visualize all the placentae in a single 3D set of slices.

Manual administration of contrast agent leads to possible variations in the input function. Although a single experienced operator took great care to keep rates constant during injection, the possibility of error due to variations in injection rate exists. In future studies, this uncertainty can be mitigated by automated injection.

We did not perform any motion correction prior to segmenting a region of interest; thus pixel shift due to breathing motion may have affected the estimated intensities and Gd concentrations.

Assumptions inherent to the reference region (RR) model (Appendix B) are difficult to justify in a mouse placenta. First, the model assumes that each compartment is uniformly mixed. This may not hold in the case of mouse placenta with two distinct flow zones present. The model assumes that the target tissue signal consists predominantly of extravascular extracellular tracer is not valid, since the target tissue signal in this case is predominantly vascular. Finally, the relatively poor temporal resolution (9 seconds) makes the estimation of rate constants with time equivalents of the same order of magnitude suspect. However, in the present case, the measured time constants are all on the order of minutes, increasing confidence in their estimation.

A shortcoming of the RR model is that the estimated placental perfusion depends on a prior knowledge of K^trans^ and K^ep^ of a selected reference tissue. The arbitrary assignment of reference tissue K^trans^ and K^ep^ values could be a source of error in the estimation of target tissue K^trans^ and K^ep^. A sensitivity analysis on the reference tissue by Yankeelov *et al*.^37^ showed that the errors in the estimation of K^trans^ and K^ep^ in the tissue of interest scale approximately linearly with the errors in the assigned K^trans^ and K^ep^ for the reference tissue. Any error in measured concentration of CA was due to fluctuations in the measured T_1_(0) that could be reflective of inherent biological variations due to differing age and health of the animal. Additionally, negative signal enhancement was occasionally observed, (corresponding to a decrease of the MR signal relative to the baseline) (Fig. 4), which could be attributed to MR signal acquisition performed during a rise in tissue temperature, consistent with temperature normalization post anesthesia induced hypothermia^40^. In these cases, voxel failures were seen where signal enhancement could not be mapped to a correct concentration.

## Conclusions

We have demonstrated for the first time that liposomal Gd particles do not penetrate the placental barrier. This could be useful in characterizing placental dysfunction where attachment of placental villi to the myometrium (inner wall of uterus) is difficult to visualize with non-contrast MRI. Perfusion kinetics of the contrast have been established, and in the case of liposomal Gd, are uncorrupted by transport across the placental barrier, and clearance kinetics, thus better reflecting the actual placental perfusion kinetics. Thus, conditions that alter the perfusion could potentially be characterized by this technique. Liposomal Gd chelates could serve as useful agents in the future to study placental architecture and boundary detection in placental anomalies such as accreta. Before use in humans however, both maternal and fetal safety of liposomal Gd will have to to be explicitly established.

## Materials and Methods

### Contrast Agents

Multihance^®^ (Bracco Diagnostics Inc. New Jersey, USA) was used as received from the manufacturer. The liposomal Gd agent (NMRX: Nano MR eXtended lifetime) was prepared in-house, following methods described in the literature^21^,^22^. Briefly, all lipids (Di Palmitoyl Phosphatidyl Choline (DPPC), Cholesterol, Methoxy Poly Ethylene Glycol - Di Stearoyl Phosphatidyl Ethanolamine (MPEG-DSPE), and Gd-DTPA- *bis* stearoyl amine (Gd-DTPA-BSA) were dissolved in ethanol in a 30:40:5:25 mole ratio and a final molar concentration (post hydration) of 40 mM and hydrated in a Tris saline buffer containing 500 mg Gd/ml gadobenate dimeglumine, such that the ethanol constituted 10% of total volume, for 1 hour. The self-assembled liposomes were then extruded 7–10 times through 400, 200 and 100 nm Nucleopore track etch membrane in a Lipex 10 ml extruder (Northern Lipids, Vancouver, BC, Canada), the excess ethanol and unencapsulated gadobenate dimeglumine removed by 10 volume exchanges in diafiltration using a 500 kDa cutoff MicroKros module (Spectrum Laboratories, CA). Mean particle size was ~120 nm, measured by dynamic light scattering. Release of Gd from the particles was tested by incubating (at 37 °C) the particle suspension at a 1:5 dilution in phosphate buffered saline (PBS, pH 7.2) and in reconstituted bovine plasma (Sigma-Aldrich, St. Louis, MO, USA) in a 100 kDa dialysis bag suspended in PBS. The external buffer was sampled at 1, 2, 24, 29, 48, and 52 hours after initiating the incubation, and analyzed for total Gd content by ICP-MS on a Varian 810 ICP-MS system. 3 batches of the nanoparticle contrast agent were tested in this manner, and each time point sampled and analyzed in triplicate. Supplemental Fig. S3 shows the results of the leakage study, and demonstrates that ~1% of the Gd appears to have leaked at 52 hours in saline, and ~4% in plasma. The DTPA chelator used in these particles has a relatively low binding constant compared to contemporary macrocyclic chelators. Use of macrocyclic chelators will therefore probably reduce this leakage even further.

### Animal models

All animal studies were performed under a protocol approved by the Institutional Animal Welfare Committee of the Baylor College of Medicine. The studies reported in this paper are in accordance with the NC3RS ARRIVE guidelines.

25 female Balb/c mice (7–9 weeks old; average weight 26 grams before pregnancy) were used in this study. Animals were mated with same strain males. The day when a vaginal copulation plug was detected was designated as day 0.5. Pregnant mice were imaged at day 16.5 ± 1. Mice were sacrificed (under the institutional euthanasia protocol) immediately after MRI data collection (except for two mice that received the liposomal Gd agent, and were maintained alive for 72 hours, allowing additional time for possible transport of Gd to the fetus, and then sacrificed). Mice were dissected and the placentae and fetuses removed and transferred individually to 2 ml polycarbonate vials for storage at −81 °C. These placentae and fetuses were analyzed using ICP-MS for the direct measurement of Gd concentration^41^.

Animals were anesthetized with 4% Isofluorane/air and transferred supine to the animal-imaging cradle. A 28-gauge catheter was placed in the tail vein. Once transferred to the imaging instrument, isofluorane (2%) was administered continuously via a nose cone. A pressure sensitive pillow was placed on the abdomen and taped in place to monitor respiration. The animal chamber of the imaging probe was maintained at around 30–33° C. The magnet room was maintained at 25–27 °C. Heart rate was maintained at steady 50–60 beats-per-minute by adjusting the airflow and isofluorane concentration. MR imaging was performed with Multihance^®^ in 14 mice. Liposomal Gd was used in 9 mice. Two mice were injected with saline as a control for comparison. Contrast agents were diluted to 20 mM and a dose of 0.08 mmol/kg (typically around 100 μL) were prepared for this study. The contrast agent was injected manually by the same operator (3 years of experience with small animals) and at the same rate in each mouse.

### DCE-MRI studies

While the liposomal contrast agent has slow clearance, Multihance^®^ clears rapidly, and timing the image acquisition is critical. We therefore opted to use the DCE-MRI technique to collect a series of images, from which the optimal time point could be selected. DCE-MRI experiments were performed on a horizontal bore 9.4 T Bruker Avance III, 20 cm bore Biospec Spectrometer (Karlsruhe, Germany) with a micro-imaging probe capable of generating gradients of (1000 mT/m). Prior to the *in vivo* study, the relaxivity of both agents was measured using the variable flip angle technique described in Appendix A. Similarly, the baseline relaxation rates, T_1_(0), for placenta and fetus were measured using the same pulse sequence as described in Appendix A.

For the DCE-MRI study, baseline images without contrast enhancement were acquired, followed by a series of images acquired over time during and after the arrival of the contrast agent in the tissue of interest. A 3D-spoiled gradient recalled echo (SPGRE) pulse sequence with a fixed flip angle of 16 degrees was used over 175 measurements. Other parameters are described in Appendix A. The time for a single measurement was 9.25 seconds. Two minutes into the experiment, contrast was injected. Total scan time was 26 minutes and 52 seconds. The acquired signal was used to generate a time-intensity curve and the signal was converted pixel-by-pixel into concentration of the injected agent.

### ICP-MS

Wet tissue from placentae or pups was chopped with a scalpel into ~3 mm pieces. A known mass of chopped tissue was then dissolved in 2–4 ml of concentrated nitric acid (14N) and heated at 100 °C till tissue completely dissolved. (Usually, about 30–45 minutes at room temperature and 3 minutes at 100 °C were sufficient). The acid solution was diluted with 10 ml of DI water, causing some precipitation of organic material. The material was centrifuged at 3000 g for 5–10 minutes, and the supernatant used for analysis. Serial dilutions over a 10^6^ range were then made, and spiked with an internal standard of Bi 10 parts per billion (ppb) in 2% nitric acid. ICP-MS analysis was conducted on a Varian 810 ICP-MS system, using the manufacturer’s recommended protocol for Gd assay. A spike-recovery study on blank tissue yielded linear response with >90% recovery over a range of 0–900 ppb and >96% response over a range of 0–50 ppb.

### Data analysis

#### Image interpretation

Due to relatively fast clearance of the Multihance contrast agent from circulation, image intensity peaked about 11–15 seconds after injection. Images from both multihance and liposomal-Gd were compared by a Radiologist to assess its quality and appearance. The images of each placenta in the field of view representing the maximum intensity achieved, were selected for reading. Images collected with the liposomal agent had constant intensity due to the very slow clearance from circulation, so an image from the series was randomly selected. Images were de-identified so the contrast agent used was not revealed, their order randomized, and presented to a blinded reader (pediatric radiologist specializing in maternal-fetal imaging). The reader rated each image on a 5-point scale (0: Placenta not visible, upto 4: clearly visible and margin delineated, comparable to major blood vessel). The scored images were then unblinded and the discrete distribution of scores for images acquired with each contrast agent tabulated. The chi-squared test was used to test the null hypothesis H0: the distributions are not equivalent.

#### DCE analysis

MRI data was transferred to an offline workstation and each placental and fetal compartment was segmented as a separate region of interest (ROI) using AMIRA V5.6 software (FEI Visualization Sciences Group, Hillsboro, Oregon). The segmented regions were transferred to Matlab (Matlab 7.6.0, MathWorks, Natick, MA, USA) to create a pixel-by-pixel T_1_(0) map and T_1_(t) map as described in Appendix A. Two placental zones were identified visually from the signal enhancement pattern: the labyrinth zone characterized by early flow enhancement and a peripheral region (maternal decidua) characterized by late flow enhancement that began after the central labyrinth enhancement peaked. The two zones together constitute the entire placenta.

DCE estimates were made for two cases: (1) assuming the placental labyrinth alone as the target tissue, and (2) assuming the entire placenta as the target tissue. A reference region (RR) model was used to estimate transfer rate constants K^trans^ and K^ep^ for the target tissue,(details in Appendix B). For both cases, a reference region in the paraspinal muscle was used, with assumed values of K^trans^ = 0.2 ml/min/ml tissue and K^ep^ = 0.1 ml/min/ml. An estimated concentration-time curve was obtained pixel-by-pixel using methods described in Appendix A.

## Additional Information

**How to cite this article**: Shetty, A. N. *et al*. A liposomal Gd contrast agent does not cross the mouse placental barrier. *Sci. Rep.* **6**, 27863; doi: 10.1038/srep27863 (2016).

## Supplementary Material

Supplementary Information

Supplementary Movie 1

## Figures and Tables

**Table 1:** Placental visibility as rated by a blinded reader (trained maternal-fetal radiologist).

Placenta Visibility	Number observed with Conventional Gd (Multihance)	Number observed with Liposomal Gd (NMRX)
1	1	1
2	6	7
3	25	17
4	15	21
5	7	1

The Chi-squared test on the score distributions indicated a probability of 2 × 10^−8^, testing the null hypothesis that the two distributions are different, suggesting that the two contrast agents yielded equivalent images.

**Table 2:** Placental and fetal concentration of gadolinium peak from MRI data.

Contrast agent	Placental concentration (μmol/L)	Fetal concentration (μmol/L)
Multihance^®^ (n = 52)	0.60 ± 0.51	0.027 ± 0.01
Liposome Gd (n = 26)	1.12 ± 0.92	Not Detectable
Control (Saline only)	Not Detectable	Not Detectable

Mean concentration using estimated T1_0_ and Eqs. A4 and A5. ROI was placed around the entire placenta and fetal sac.
