# Corrigendum: A liposomal Gd contrast agent does not cross the mouse placental barrier

**DOI:** 10.1038/srep39724

**Published:** 2017-02-16

**Authors:** Anil N. Shetty, Robia Pautler, Ketan Ghaghada, David Rendon, Haijun Gao, Zbigniew Starosolski, Rohan Bhavane, Chandreshkumar Patel, Ananth Annapragada, Chandrasekhar Yallampalli, Wesley Lee

Scientific Reports 6: Article number: 2786310.1038/srep27863; published online: 06 14 2016; updated: 02 16 2017

The original version of this Article contained a typographical error in the spelling of the author Ketan Ghaghada, which was incorrectly given as Ketan Ghagahda. This has now been corrected in the PDF and HTML versions of the Article.

Additionally, the original Supplementary Information file published with this Article did not contain a reference list. This has been corrected in the Supplementary Information that now accompanies the Article.

