# Supplementary material for: A liposomal Gd contrast agent does not cross the mouse placental barrier
Source: Sci Rep. 2016 Jun 14;6:27863. doi: 10.1038/srep27863 (PMC4906290; doi:10.1038/srep27863)
Supplement: Supplementary Information [file srep27863-s1.pdf]

SUPPLEMENTARY MATERIAL

A liposomal Gd contrast agent does not cross the mouse placental barrier.

Anil. N. Shetty, Ph.D.[1], Robia Pautler, Ph.D.[2], Ketan Ghagahda, Ph.D.[3] ,David Rendon, Ph.D.[1], Haijun Gao, Ph.D.[1], Zbigniew Starosolski, Ph.D.[3], Rohan Bhavane, Ph.D.[3], Chandreshkumar Patel, M.S.[3],Ananth Annapragada, Ph.D.[3], Chandrasekhar Yallampalli, Ph.D.[1], Wesley Lee, M.D.[1]

[1]Department of Obstetrics and Gynecology

[2]Department of Molecular Physiology and Biophysics

[3]Department of Pediatric Radiology Texas Children's Hospital

Houston, Texas

APPENDIX A:

Phantom study

Phantom experiments were performed to establish CA relaxivity at the operating field of 9.4T. A spoiled gradient recalled echo (SPGRE) sequence is frequently used in gadolinium chelate-based studies of spin-lattice relaxation time constant (T1)-weighted DCE-MRI. These pulse sequences are optimally T1-sensitive and minimally spin-spin relaxation time constant (T2)/ apparent spin-spin relaxation time constant (T2*) sensitive [38-40]. The pulse sequence can be used either in a 2D or a 3D mode and can be implemented with sufficiently high temporal resolution that is adequate to characterize the CA transit. A series of vials containing contrast agents with concentrations (0.2, 0.25, 0.40, 0.50, 0.60, 0.75, 0.80, 1.0 mmol) was constructed to estimate T1. A 3D-SPGRE pulse sequence with a series of RF pulse flip angles (16^0, 23^0, 35^0, 40^0, 54^0); repetition time (TR) 6 ms; echo time (TE) 1.93 ms; single average, spectral bandwidth 98.68 KHz, field-of-view (FOV) 3.0 x 3.0 x 2.5 cm, matrix 128 x 128 x 16, voxel resolution 0.234 x 0.234 x 1.562 mm^3 was used. The receiver gain was held at the same level between flip angle changes. Signal in each pixel following a gradient recalled pulse sequence with at a flip angle (α_i); pulse repetition time (TR) and pulse echo time (TE) can be written as [41];

$$S_{\alpha_i} = \frac{M_0(1-e^{-TR\cdot R1})e^{-TE\cdot R2}}{(1-Cos\alpha_i\cdot e^{-TR\cdot R1})}Sin\alpha_i \approx S_{\alpha_i} = \frac{M_0(1-e^{-TR\cdot R1})}{(1-Cos\alpha\cdot e^{-TR\cdot R1})}Sin\alpha_i \qquad \text{(A1)}$$

Here, M_o includes instrument related parameters and the total proton density and is assumed to be the same between measurements at different flip angles. In T1-weighted imaging, with TE <<T2 , $e^{-TE\cdot R2}\sim 1$ terms involving R2 (=1/T2) are ignored.

Upon simplification,

$$\frac{S_{\alpha_i}}{Sin\alpha_i} = \frac{S_{\alpha_i}}{Tan\alpha_i}\cdot E_1 + C. \qquad \text{(A2)}$$

Plotting $\frac{S_{\alpha_i}}{Sin\alpha_i}$ vs. $\frac{S_{\alpha_i}}{Tan\alpha_i}$, the intercept M_0 $(1-e^{-TR/T1})$ and slope E1 (= $e^{-TR/T1}$) can be estimated at each concentration, from which T1 = -TR/Ln (E1) is estimated. The slope of (1/T1) vs. Concentration plot (Figure S1) is the *relaxivity*.

Pre- and post Gd T1 estimation

Prior to post-Gd dynamic imaging, a series of localizers were obtained using three-plane spoiled gradient recalled echo sequence to visualize fetal anatomy. A single 3D-SPGRE sequence was used with identical parameters and orientation for both T1(0) and T_1 (t) estimation. Pulse sequence parameters were: TR =6 ms, TE = 1.93 ms, Flip angle = 16 degrees, effective bandwidth = 98.68 kHz, FOV = 35 mm x 35 mm x 20 mm, with effective partition thickness = 1.25 mm, matrix = 128 x 128 interpolated to 256 x 256 with 20 dummy prep-scans, and effective scan time for single measurement = 9.215 secs. For dynamic imaging, the number of measurements were increased to 175 making the total time for imaging of 26 mins and 52 secs. The contrast agent was injected 2 minutes into the measurement. If the bulk magnetic susceptibility (BMS) shift is negligible, the presence of paramagnetic center enhances proton relaxation rate and the relationship between tissue relaxation time (T_1) following the administration of CA is determined by the Solomon-Bloembergen equation that relates relaxation rates as [42,43]:

$$\frac{1}{T_1(t)} = \frac{1}{T_{10}} + r1\cdot CA(t) \qquad \text{(A3)}$$

where T1(0) is the T_1 value before CA administration (native relaxation time) and r_1 is the longitudinal relaxivity of the administered agent. Since relaxation rates are additive, one can write the above equation as:

$$R_1(t) = R_{10} + r_1\cdot CA(t) \qquad \text{(A4a)}$$

$$CA(t) = \frac{1}{r_1}[R_1(t) - R_{10}] \qquad \text{(A4b)}$$

where relaxation rate (R) is the reciprocal of the relaxation time. In biological tissues the above relationship may not hold exactly due to heterogeneity and compartmentalization within each MR voxel. The post contrast T_1(t) is estimated by using the following equation [44]:

$$\frac{1}{T_1(t)} = -\frac{1}{TR} \cdot \ln \frac{\left[\frac{S_\alpha(t)}{S_\alpha(t=0)}\left(e^{\frac{TR}{T_{1,0}}}-1\right)-e^{\frac{TR}{T_{1,0}}}+Cos\alpha\right]}{\left[\frac{S_\alpha(t)}{S_\alpha(t=0)}\left(e^{\frac{TR}{T_{1,0}}}-1\right)Cos\alpha-e^{\frac{TR}{T_{1,0}}}+Cos\alpha\right]} \quad \text{(A5)}$$

where $S_\alpha(t)$ refers to measured signal at a flip angle α and a time t, $S_\alpha(t=0)$ is the baseline signal and $T_{1,0}$ is the endogenous relaxation time prior to injection. Once having measured T_1 (t), T1 (0) and r_1 and upon substituting in Eq. (A4b), CA was calculated pixel-by-pixel on the segmented ROI, and integrated over the ROI to yield overall values. Movie1 shows typical dynamic study with contrast uptake in placenta.

APPENDIX B:

Reference Region model

The most commonly used compartmental model for measuring pharmacokinetics is described by Tofts et al. [45,46] and incorporates the exchange of CA between plasma (extracellular vascular space) and tissue of interest. The input for this model is based on the knowledge of flux or concentration in each compartment as a function of elapsed time. The rate of transfer, or *flux* from the vascular space to the tissue space can be described by the volume transfer constant K^{trans}, while the efflux rate of CA, K^{ep}, moving from tissue space back into vascular space is defined as K^{trans}/v_e. For a simple two compartment construct represented by plasma and tissue the differential form for kinetics is given by [45];

$$\frac{d}{dt}C_{tis}(t) = K^{trans.tis} \cdot C_p(t) - K^{ep.tis} \cdot C_{tis}(t) \quad \text{(B1)}$$

where, $C_{tis}(t)$ and $C_p(t)$ are the CA concentrations in the tissue space and the plasma space, respectively and $K^{ep.tis} = K^{trans.tis}/v_{e.tis}$. The concentration of the CA in blood vessels C_{blood} is related to plasma through the hematocrit (Hct) as C_b (t) = C_p(t). (1-Hct) where Hct is estimated to be 0.45 in mice [47]. The solution to eqn. (B1) is given by [45]:

$$C_{ti?}(t) = K^{trans.tis} \int_0^T C_p(t) \cdot e^{-K^{ep.tis}(T-t)}\, dt \quad \text{(B2)}$$

The above equation neglects the vascular volume in the tissue itself. For normal tissues with low vascular fractions (< 2%) this is a reasonable assumption. In order to employ Tofts model based on Eqns. (B1 and B2), the rate of change of contrast agent concentration in both vascular space and tissue must be known. The vascular contrast agent concentration is the vascular input function (VIF) and in small animals it is difficult to estimate due to poor temporal resolution. Additionally, due to small field of view, major blood vessels are often outside the imaging field of view.

Because of the difficulty in estimating VIF, an alternative model called "reference region" (RR) model was used. Analysis by the RR method utilizes a well-characterized tissue such as muscle to calibrate the signal in the ROI. Using pair-wise compartments with common plasma compartment, one can eliminate plasma compartment completely, thus eliminating the need to characterize VIF. The advantage of this model is that it is not necessary to have blood vessel in the FOV or to directly measure VIF. The relevant equation governing reference tissue and plasma would be similar to Eq. (B1):

$$\frac{d}{dt}C_{RR}(t) = K^{trans.RR} \cdot C_p(t) - K^{ep.RR} \cdot C_{RR}(t) \quad \text{(B3)}$$

Where $K^{trans.RR}$ and $K^{ep.RR}$ are the appropriate quantitative parameters for the reference region tissue, C_{RR} is the measured concentration of CA in the reference region tissue, and C_p is the plasma concentration of the blood. Eqs. B1 and B3 allow for the elimination of C_p, and the solution of the resulting differential equation is given by [34]:

$$C_t(t) = \frac{K^{trans.tis}}{K^{trans.RR}} \cdot C_{RR}(t) + \frac{K^{trans.tis}}{K^{trans.RR}} \cdot \left[K^{ep.RR} - K^{ep.tis}\right] \cdot \int_0^t C_{RR}(\tau) \cdot e^{-(K^{ep.tis})(t-\tau)} d\tau \quad \text{(B4)}$$

where $K^{ep.tis} = \frac{K^{trans.tis}}{v_{e.tis}}$ and $K^{ep.RR} = \frac{K^{trans.RR}}{v_{e.RR}}$. An estimation of the plasma concentration of CA was obtained using paraspinal muscle as a reference region with fixed values of $K^{trans.RR}$ and $K^{ep.RR}$ [48]. The solution of Eq. B4 is based on model fitting in which $C_t(t)$ and $C_{RR}(t)$ are concentrations in target- and reference-tissues respectively. These concentrations are estimated by using Eqs. A4b and A5. The model fitting is based on Levenberg-Marquardt algorithm (LMA) to perform the non-linear least squares regression in each pixel of the analyzed region of interest (ROI). LMA has been successfully used in the pharmacokinetic modeling of DCE-MRI [49]. The RR model fitting was

performed using the software developed by Ortuno et al [50]. A public release of the software and open source code is available at http://www.die.upm.es/im/archives/DCEurLAB. The model yields K^{trans} and K^{ep} in units of min^{-1} that can be used to express flow as ml/min/ml of tissue. Figure S2 shows modeled and acquired DCE–MRI profiles for a selected placental ROI. The dots represent calculated contrast concentration based on Eq. A4, while the solid curve is the best fit RR model.

APPENDIX C:

Gadolinium assay

Following MRI study, animals were euthanized according to the institutional guideline and dissected to extract the placenta-fetal sac. From these excised sacs, placentae and fetuses were randomly selected for Multihance® (n = 8) and Liposomal Gd (n = 6), separated and placed in a vial and stored at -81^{0}C for later Gd analysis using ICP-MS (Inductively Coupled Plasma Mass Spectrometry: Nexion 300 or a Varian 810-MS). Analysis was performed according to the procedure described by Frame et al [37]. Frozen whole fetus and placenta samples were thawed at room temperature and wet tissue weight was recorded. The tissues were then dissolved in concentrated nitric acid (90%) in borosilicate glass scintillation vials. 4 ml and 2 ml of acid were used for each fetus and placenta samples respectively. The acid tissue mix was heated over a water batch at 100^{0}C for 2-5 minutes till all tissue was dissolved. All acid handling and manipulation was done in a fume hood. The acid dissolved tissue was then diluted with 10 ml of DI water, the volume after dilution was measured and the contents were transferred to a centrifuge tube. The tubes were then centrifuged at 3000g for 5-10 minutes to separate tissue precipitates formed after dilution. The clear supernatant was then further diluted with DI water 3x, 10x and 100x. Clear samples were not treated further. Samples that either formed additional precipitates or turned hazy were then filtered through 0.2 µm Millex GP (PES membrane) syringe filter. Bismuth nitrate was added at a final concentration of 10 ppb in all samples as an internal standard. The final concentration of nitric acid ranged from 1-6 %. In order to estimate recovery of Gd due to the various treatments, a blank tissue sample of fetus and placenta (no Gd treatment) was spiked with a known amount of Gd and the treatment procedures as above were followed. Final Gd recovery, as detected by ICP-MS, was between 90-92 %. Since liposomal Gd has a circulation half-life of 18 - 24 hours, assay experiments with liposome Gd was performed after 72 hours in two animals to rule out any contrast uptake in the fetal compartment over time.

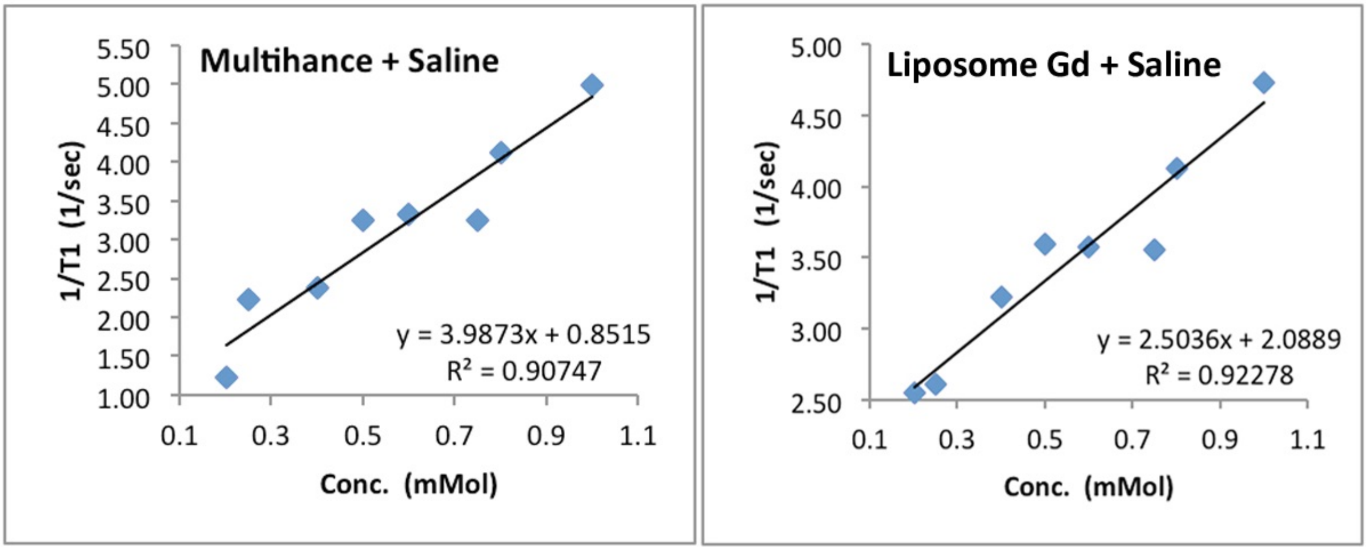

Figure S1.Relaxivity measurement. Plot of relaxation rates of Multihance® and liposome Gd agents at increasing concentrations in saline yields a slope equal to the molar relaxivity (sec-1mMol-1).

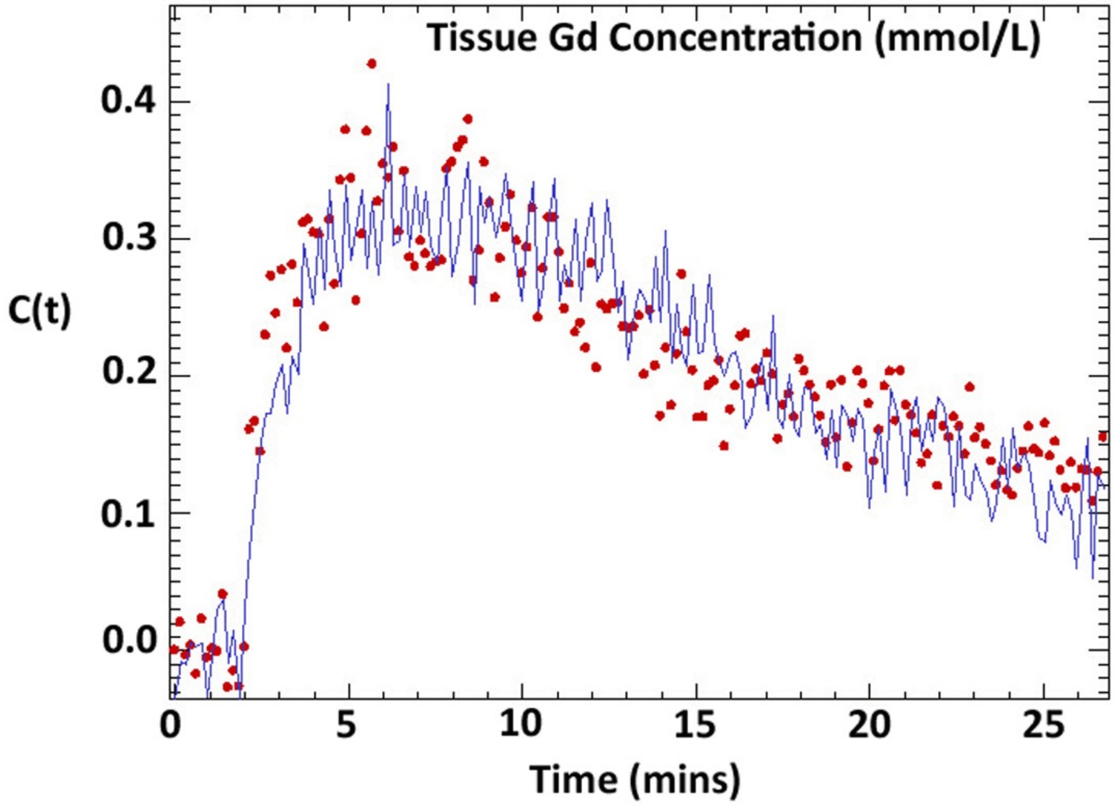

Figure S2. Modeled and acquired DCE-MRI profiles. Modeled and acquired DCE-MRI profiles in one placenta. Red dots represent the estimated concentrations (mM) based on Eqs. A4 and A5 and the Blue line represents the best-fit Reference Region model. The fitted parameters Ktrans,Kep and Ve for this instance are shown on the right.

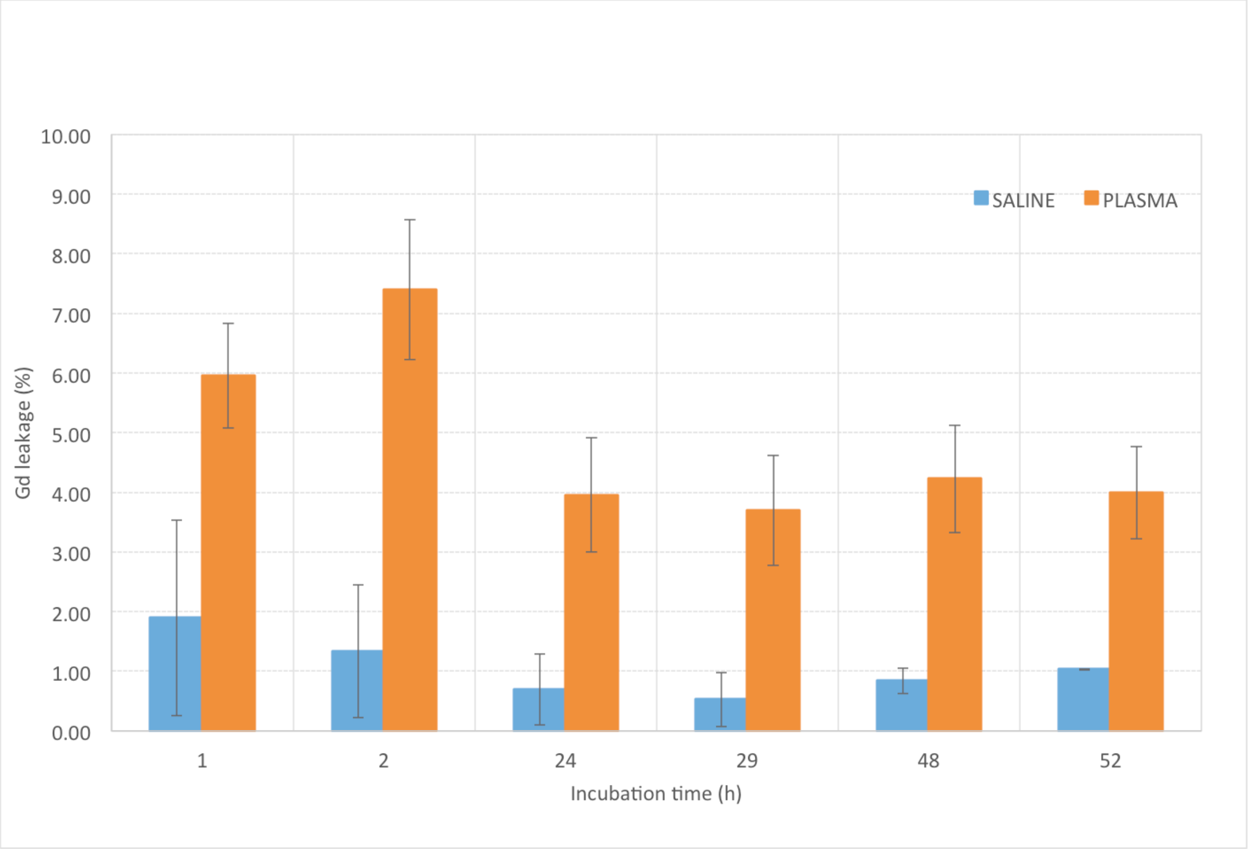

Figure S3. Leakage of Gd from liposomal nanoparticles in Saline (blue bars) and reconstituted bovine plasma (orange bars).

Please see attached movie file

Movie1. DCE-MRI. Dynamic images of mouse placentae following the injection of a Multihance® contrast agent. The placenta shows early filling of central labyrinth followed by transitional and peripheral zones. Each frame of the movie corresponds to one time point in the DCE-MRI acquisition, ~9.25 seconds.
